# Transcatheter Aortic Valve Implantation (TAVI) in Bicuspid Anatomy

**DOI:** 10.3390/jcm14030772

**Published:** 2025-01-24

**Authors:** Dimitrios N. Nikas, Lampros Lakkas, Katerina K. Naka, Lampros K. Michalis

**Affiliations:** 1 1st Cardiology Department, Ioannina University Hospital, 455 00 Ioannina, Greece; 2 Department of Physiology, Ioannina Medical School, 455 00 Ioannina, Greece; 3 2nd Cardiology Department, Ioannina University Hospital, 455 00 Ioannina, Greece; anaka@uoi.gr (K.K.N.); lamprosmihalis@uoi.gr (L.K.M.)

**Keywords:** TAVI, aortic stenosis, bicuspid

## Abstract

Bicuspid aortic valve (BAV) stenosis, a common congenital condition, presents unique challenges for transcatheter aortic valve replacement (TAVI) due to anatomical variations like cusp morphology, coexisting aortopathy and calcification. TAVI offers a viable option for BAV patients with refinements in technique and technology, though ongoing research is essential to optimize patient-specific approaches and long-term results. Key considerations for TAVI in BAV include precise valve sizing, positioning, and the need for rigorous pre-procedural imaging to mitigate risks such as paravalvular leak and stroke. Early results show TAVI’s safety and efficacy are comparable to surgery, though BAV patients undergoing TAVI often are exposed to higher rates of post-procedural pacemaker implantation. Emerging data on next-generation self-expandable (SE) and balloon-expandable (BE) valves reveal that while both offer success in this complex anatomical aortic valve variation, gaps remain in the long-term durability and management of BAV-related aortopathy. This review examines the latest advancements in TAVI for BAV, emphasizing how specialized approaches and device selection address BAV’s complexities.

## 1. Epidemiology

Bicuspid aortic valve (BAV) is the most common congenital disease of the heart, observed and reported over 400 years ago by Leonardo da Vinci [1]. The estimated prevalence of BAV in the general population is estimated to be between 0.5 and 0.8% [2].

## 2. Anatomy and Variations

The normal tricuspid aortic valve has a simple anatomy (since every other variation should be considered abnormal), consisting of three cusps of semilunar shape, triangles between cusps, and three Valsalva sinuses. Conversely, BAV has a different anatomy with many variations, thus creating a broad spectrum of morphological entities, from two cusps, two intercusp triangles, and two Valsalva sinuses, to morphologies with three Valsalva cusps and a raphe (which is an aborted inter-cusp commissure during embryonic development) that divides one cusp into two parts [3]. Another commonly used classification has been proposed by Sievers [4]. This classification system is based on three important anatomic and functional characteristics: (1) the number of raphes, (2) the spatial position of cusps or raphes, and (3) the functional status of the valve. This classification was primarily introduced to provide an easy and comprehensive means of scientific communication in order improve treatment. According to this, three major types of BAV anatomy were identified: type 0 (no raphe), type 1 (one raphe), and type 2 (two raphes). This was followed by two supplementary characteristics, namely spatial position and function. According to this classification, six (6) different types of BAV have been found to have raphes with fusion between one or more cusps, either in the left or/and right cusp. 

**Figure 1:**
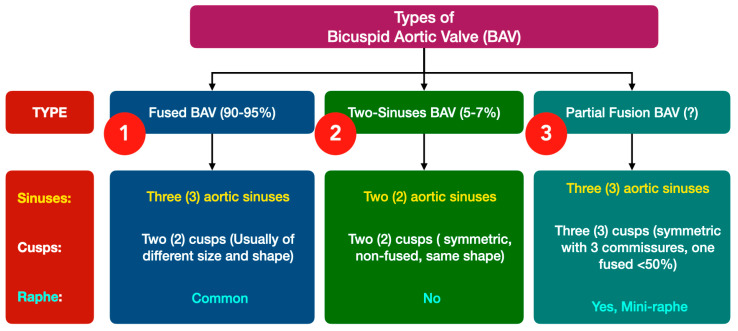
Suggested classification of the BAV anatomy (modified by Michelena et al., Ref. [4]): The consensus proposes a relatively simple classification according to three (3) components of the anatomy: sinuses, cups and the presence or absence of raphe. (“?” Indicates that incidence of the cases are not precisely known).

Although various BAV classification systems have been proposed in recent decades, a novel international consensus is currently used to better understand the subtypes of BAV. It is based on various characteristics, mainly consisting of valve function, raphe anatomy, cusp anatomy and the presence of aortopathy. This classification system proposes three types of BAV with various sub-phenotypes. In overall, it has many advantages over previous ones: (a) it can depict all various BAV morphologies, (b) it includes different aorta phenotypes, (c) it inherently includes symmetry assessment (suitable for surgical repair planning), and (d) it recognizes rare BAV phenotypes, such as fused BAV without raphe and three Valsalva sinuses [5] (Figure 1).

## 3. The Valve–Aorta Disease

BAV is a far more complex disease than valvular heart disease itself, since it also includes disease of the ascending aorta. There are two distinct features of this conundrum: (A) BAV can be an inherited disease, with familial predisposition and degenerative changes in the aortic wall, due to fibrillin deficiency and the release of certain metalloproteinases that cause damage to the tunica media of the aorta. In addition, (B) the presence of BAV creates flow abnormalities, with asymmetrical patterns with several type of turbulence [6]. This setting may cause increased wall stress in the ascending aorta, thus increasing the relative risk of dilatation. The main dilemma and clinical question at this point is whether aortopathy in patients with BAV comes as a part of congenital heart disease or as an expected flow-related local phenomenon [7]. Moreover, patients with BAV usually present with other congenital heart diseases such as ventricular septal defect, aortic arch obstruction and aortic arch coarctation, making things even more complicated, as these complex cases may need a different approach in terms of diagnosis and treatment [8].

## 4. Natural History

It is known that patients with BAV are more likely to have calcific aortic stenosis (AS) than patients with a tricuspid aortic valve, due to the specific anatomic and histological characteristics [9]. Several factors may contribute to this observation. Abnormal hemodynamics along with altered turbulent flow and localized cusp stress may lead to endothelium damage, inflammation and calcification over time. The presence of fusion between the cusps may lead to less flexible cusps and therefore more make individuals more prone to micro-ruptures and future calcification [10]. In addition, genetic predisposition and inflammatory responses may contribute to more intense cusp calcification, stimulating a calcification cascade that typically increases with age [11,12]. Previous reports have shown that AS in BAV is associated with greater levels of cusp inflammation and neo-vascularization, especially on the aortic side compared to the left ventricular outflow tract side of the valve. Even though there have been many speculations about increased shear stress, the presence of intracusp hemorrhagic predisposition along with potential genetic mutation is an independent predictor of AS in BAV patients. Moreover, the greater incidence of fibrotic plaques in BAV-related AS cases might be an acquired and not inherited response to chronic cusp damage, due to turbulent flow [13]. This explains why patients with BAV may develop AS needing intervention at a younger age (at least ten years earlier).

In a large cohort of young, ambulatory patients (*n* = 642) with BAV who were followed up for a period of nine (9) years, one-quarter (25%) of them had one primary cardiac event, either cardiac death, intervention on the aortic valve or ascending aorta, aortic dissection or aneurysm, or congestive heart failure requiring hospital admission. In this study, the severity of AS or AR was independently associated with primary cardiac events [14]. The long-term outcome of BAV patients has been shown to vary according to the clinical/echocardiographic stage of the disease and the age of the patient. In younger, and thus at a non-severe stage of AS, the natural history of the disease is almost equal to the general population with tricuspid aortic anatomy, whereas in the older population or those with more severe stenosis, the long-term outcome is significantly impaired [15]. In younger ages, BAV-associated morbidity is higher in men while in advanced ages and in a state of stenosis, women exhibit a higher risk of death, independently associated with the presence of AR. Men present more frequent aortopathy than women, mainly due to genetical, hormonal and possible higher hemodynamic stress [16,17].

All the above state that BAV significantly affects patients’ outcome, meaning that the long-term survival of those patients is considerably impaired, and therefore early diagnosis and intervention may be required.

## 5. Recommendations for Intervention

In patients with BAV-related AS without concomitant aortopathy, the indications for valve intervention are the same as those for native tricuspid aortic valves patients, according to both European and US guidelines for valvular heart diseases [18,19]. This means that the decision to perform valvular intervention should be made according to symptoms, systolic function and the internal diameters of the left ventricle, or when a concomitant indication for other cardiac interventions (i.e., coronary artery bypass graft surgery) exists. In the case of BAV-related aortopathy, suggestions are different in guidelines, but the presence of additional risk factors [(family or individual’s history of aortic dissection, severe AR, severe mitral regurgitation, desire for pregnancy, systemic hypertension and aortic size increase > 3 mm/year according to the European guidelines), (family history of aortic dissection, aortic growth rate > 5 mm/year for US guidelines)] is generally regarded as an indication for intervention.

A maximal ascending aorta diameter > 55 mm (measurements confirmed by ECG-gated CT) should be treated surgically (IIaC in European guidelines, I in US guidelines). If there are any of the additional risk factors, the cut-off diameter for the ascending aorta is >50 mm (IIa for both guidelines). When BAV-related AS and concomitant aortopathy is present, double surgery is an acceptable approach (IIa for both guidelines), with an aortic root dilated ≥ 45 mm. Moreover, both guidelines suggest that an (a) indexed aortic diameter should be used, with a cut-off value ≥ 25 mm/m^2^ used as an accepted limit for surgical treatment; (b) valve-sparing surgery may be considered for patients with BAV-related aortopathy and eligible for surgical treatment without at least moderate stenosis. Many recent studies have shown that BAV-related aortopathy is worse, in terms of symptoms and disease progress, in patients with BAV-related regurgitation, rather than stenosis [20,21].

## 6. Current Treatment Strategies

### Surgery

Surgical aortic valve replacement is the preferred treatment for patients presenting with severe BAV stenosis with or without aortic aneurysm. Importantly, the presence of aortic aneurysm is a determinant factor for the selection of the type of surgery. In patients considered at a higher risk of surgical aortic valve replacement, the presence of a concomitant ascending aortic aneurysm may necessitate the treatment of both the valve and the aorta. In such scenarios, the complete surgical replacement of the aortic valve and aorta could be viewed as the only feasible option, depending on individual patient characteristics and clinical considerations.

Several anatomical and functional features of both the aortic valve and aorta play an important role in selecting the appropriate surgical technique. Apart from aortic dilatation, the particular type and extent of fused cusps, the prolapse of the non-fused cusps, the presence of stenosis or regurgitation, or both, and the severity of aortic annulus dilatation are all considered key factors for selecting the correct surgical procedure [22]. Recent advances have resulted in very good long-term outcomes with very low in-hospital surgical mortality. Age, the presence of aortic dilatation, and incremental degrees of AS and AR have been identified as predictive of adverse events. Valve-sparing techniques could potentially evolve significantly to offer promising acute and long-term outcomes, though not necessarily to the same extent as aortic valve replacement techniques, particularly in cases involving aortic regurgitation [23,24]. In the presence of aortic dilatation, the preservation of the aortic valve is still feasible after aortic replacement with improved durability [25,26]. Alternatively, annuloplasty can also be used in isolated BAV repair or as an adjunct to surgical valve-sparing root remodeling, with excellent 10–15-year results [27].

## 7. Transcatheter Aortic Valve Implantation (TAVI)

The history of TAVI in patients with BAV-related AS began with small case series. In 2010, Wijesinghe et al. showed results from TAVI in BAV-related AS using first-generation ballon-expandable (BE) valves in 11 patients, achieving successful deployment in 10 of them [28]. Those initial attempts were followed by larger studies, using second-generation valves. Mylotte et al., in 2014, showed a 3.6% peri-procedural mortality rate (one-year 17.5%) in 139 patients with BAV-related AS treated with second-generation BE or self-expandable (SE) valves. A considerable proportion of these patients (28.4%) developed at least moderate AR after TAVI (not defining whether it was intravalvular or paravalvular) [29]. In all early studies, the device success rates were lower in patients with BAV compared to normal tricuspid valves [9]. Earlier TAVI devices had higher rates of paravalvular leak (PVL), whereas the new ones offer better anchoring and sealing capabilities, resulting in lower rates of PVL and improved procedural success rates. Additionally, even though the results did not reach statistical significance, the lower profile of the newer devices reduced vascular complications, which could be attributed to better patient outcomes [30].

The advent of third-generation SE and BE valves with attractive technical characteristics (such as smaller delivery sheaths, wider variety of sizes, the presence of outer sealing skirts for BE valves, pericardial outer wrap for SE valves), as well as the increased use of CT-based anatomical measurements (such as aortic annulus and aortic root diameter and perimeter, coronary ostia height, coronary sinus depth, aortic angle), have also improved procedural success and reduced the rate of para-valvular regurgitation [31]. Perlman et al., in 2016, showed a high procedural success rate when using the Sapien-3 valve, a balloon-expandable valve, in 51 patients with BAV-related AS; this showed that this valve was suitable for TAVI without any significant para-valvular regurgitation at 30-day follow-up [32].

In 2020, the Society of Thoracic Surgeons and the American College of Cardiology published the Transcatheter Heart Valve Therapies registry for patients with BAV-related AS that underwent TAVI with a BE valve from 2015 to 2018 (a total of 932 patients), with a self-expandable Evolut™ or Evolut Pro™ TAVI device (Medtronic, Minneapolis, MN, USA). This prospective study showed successful implantation in 99% of cases. There was no difference in device success between BAV and tricuspid valves (96.5% vs. 96.6%, *p* = 0.87). Patients with BAV had a higher incidence of ischemic stroke (2.5% vs. 1.6%, *p* = 0.02) and a higher pacemaker implantation rate (9.1% vs. 7.5%, *p* = 0.03) at 30 days after TAVI [33]. The BEAT International Collaborative registry compared the performance of next- generation BE (Sapien-3 valve) to SE (EvolutR and Evolut Pro) valves in patients with BAV-related AS. In a total of 242 patients, there was similar device success in both the propensity score cohort and the unmatched cohort. Patients treated with BE valves received transcatheter valves of smaller size compared to the SE group (23 mm 23.4% vs. 3.9%, 26 mm 41.6% vs. 23.4%, *p* < 0.001). There were no differences in the 30-day clinical outcomes, but hemodynamic parameters favored the SE group. Importantly, a higher rate of annular rupture in the BE group was noted [34].

In 2020, Ueshima et al. presented the results of a systematic review and meta-analysis of seven studies (a total of 706 patients) comparing first- and second-generation BE and SE valves in BAV-related AS. BE valves were associated with lower rates regarding the need for a second valve (2.8% vs. 9.1%, *p* = 0.05) and new pacemaker implantation (15% vs. 22.1%, *p* = 0.05), but as it mentioned before, they were associated with higher rate of annular rupture (3.5% vs. 0%) [35]. Yoon et al. reported upon data from the International BAV Stenosis registry. In this study, 72% of the included patients had an implanted Sapien-3 (BE valve). Patients with calcified raphe and excess cusp calcification had significantly higher two-year all-cause mortality rates than patients with one or none of these features (6.5%, 2.5% and 1.6%, *p* = 0.002) [36]. In 2021, Forrest et al. published the results of the Low-Risk Bicuspid study, a single-arm prospective study that enrolled a total of 150 low-risk patients (predicted risk of 30-day mortality less than 3.0%) with BAV-related AS and treated with SE valves (Evolut™-R or Evolut™-Pro, Medtronic). A high device success (95.3%) rate accompanied by a low rate of all-cause mortality (1.3%) was observed [37]. Both studies highlight that morphological and anatomical BAV characteristics may play a role in the outcome of patients. Therefore, those patients presenting with “low-risk” features, like less raphe calcification or low surgical risk, may be considered for TAVI with an acceptable anticipated outcome, unless that surgery is excluded for whatever reasons.

There is a lack of data comparing TAVI to surgical valve replacement in patients with BAV-related AS, since BAV patients were excluded from all landmark TAVI randomized trials [3]. Elbadawi et al., in 2019, presented the results of a propensity-matched study, comparing TAVI to surgical valve replacement in 975 patients with BAV-related AS. The two main results of this study were as follows: (a) a similar in-hospital mortality (3.1% vs. 3.1%, OR 1.00, 95% CI 0.59–1.97, *p* > 0.999) and (b) no difference in adverse events. TAVI was associated with a higher rate of pacemaker implantation (13.8% vs. 4.6%, OR 3.32, 95% CI 2.34–4.71, *p* < 0.01) and a lower rate of bleeding and transfusion [38].

Recent results from the NOTION-2 trial provide significant data regarding the comparison between TAVI and surgical aortic valve replacement (SAVR) in low-risk patients aged 60 to 75 with severe symptomatic aortic stenosis [39]. The study included both tricuspid and bicuspid aortic valve anatomies. At 1-year follow-up, the primary composite endpoint of all-cause mortality, stroke, or rehospitalization occurred in 10.2% of patients in the TAVI group and 7.1% in the SAVR group (*p* = 0.3). In patients with tricuspid aortic valves, the incidence of the primary endpoint was similar between TAVI and SAVR, indicating clinical equipoise. However, in patients with bicuspid aortic valves, the primary endpoint occurred more frequently in the TAVI group (14.3%) compared to the SAVR group (3.9%), whereas the risk of stroke was higher in the TAVI group, particularly among bicuspid patients. These findings indicate that, at least in bicuspid patients, caution is warranted when considering TAVI for patients with bicuspid aortic valves due to the observed higher incidence of adverse events, especially stroke.

## 8. Need for Early Intervention

In patients with BAV, the exact timing of surgical intervention is crucial, in order to achieve the best outcome. As BAV is considered a combined disease involving both the aortic valve and ascending aorta, anatomical and functional parameters, like the diameter of the aorta or the severity of the aortic valve stenosis/regurgitation, are important to consider when selecting the appropriate time for intervention. These factors seem to play an important role in the future outcome of BAV patients. In a study population of young BAV adults, the severities of AS and AR were independently associated with primary cardiac events, without, however, affecting the 9-year survival rate of the BAV patients, which was relatively similar to the general population [14]. Furthermore, other important factors like continuous aneurysm progressive dilatation (>5 mm/year) or the functional worsening of the aortic valve—either stenosis or regurgitation—are also regarded as reasons for early intervention in these patients [40]. The post-surgery outcome post is excellent in these patients, and not different to that expected for the overall population, regardless of age [41]. In general, patients presenting with BAV may experience earlier surgical interventions mainly due to the presence and progression of the disease at a younger age. However, any decision to perform surgical intervention should be carefully weighed against the periprocedural complications and the long-term durability of the prosthetic valve.

## 9. Reasons for TAVI

As life expectancy increases, more BAV patients may develop aortic stenosis later in life, potentially making TAVI a viable option, especially for older individuals with comorbidities [2,42]. A study by Roberts et al. reported that 22.2% of patients aged ≥ 80 years with surgically excised stenotic aortic valves had congenitally stenotic BAVs [43]. The shorter hospital stay and quicker recovery associated with TAVI could also make it appealing to patients seeking rapid rehabilitation [44]. With expanding indications for TAVI, it is possible that younger BAV patients may increasingly be considered for the procedure. However, several drawbacks still need to be addressed and possibly changed in order for TAVI to become the first-line therapy for BAV patients. Until then, surgery will remain the best option for treatment for those BAV patients presenting with severe aortic stenosis.

## 10. Implantation

The bicuspid aortic valve is a special type of AS, presenting with several anatomical types that are different to the ones of the tricuspid. Therefore, specific implantation techniques are needed to achieve results comparable to that of the tricuspid AS. The successful implantation of a transcatheter aortic bioprosthesis in a bicuspid anatomy depends on these important steps: detailed pre-procedural planning, correct BAV sizing, and accurate implantation techniques.

In pre-procedural native valve evaluation, Multi-Slice Computer Tomography (MSCT) regards the “gold standard” as measuring the dimensions and size of the native aortic valve. In BAV patients, the detailed and accurate measurement of all anatomical parameters is essential for the success of the implantation. The proper type and size of the bioprosthesis has been proven to improve procedural results and long-term valve performance. Therefore, meticulous valve sizing is crucial for the appropriate selection of bioprosthesis. In all BAV anatomies, additional measurements need to be made in order to estimate both the size of the bioprosthesis and the landing zone, which might be different to that of a normal aortic valve. Accurate valve sizing and meticulous positioning ensure the proper expansion of the valve.

Sizing in BAV anatomy may be particularly difficult. In a tricuspid anatomy, the smallest perimeter, and therefore the point at which the valve should be positioned to achieve adequate anchoring and sealing, is the aortic annulus. However, in BAV cases, the smallest and most appropriate point for the implantation of the bioprothesis may be higher than the anatomical annulus. As a result, additional measurements may be required to select the appropriate level of implantation and the size of the bioprosthesis. Using the large BAVARD (Bicuspid Aortic Valve Anatomy and Relationship With Devices) retrospective registry, Tchetche et al. aimed to capture the sizing ratios used for transcatheter aortic valve implantation in BAV and analyze the second-generation prostheses geometry post-implantation. An intercommissural distance (ICD) of 4 mm above the annulus, compared to the anatomical annulus dimensions, has been proposed to define a tubular, flared or tapered aortic anatomy [45]. Even though there is no clear justification, the authors set the ICD distance as 4 mm above the annulus, for simplification and standardization. According to this configuration, different sizing levels should be used: in the tubular and flared type, the regular annulus perimeter/area should be used, while in a tapered anatomy, the supra-annular perimeter/area 4 mm above the anatomical annulus should be used as a reference to select the appropriate transcatheter bioprosthesis [46]. However, new data indicate that, even when using this method, no incremental benefit may be observed with regard to the final choice and the outcome of the device [47].

Several anatomical features specific to BAV should be taken into account when selecting the size and type of transcatheter bioprosthesis. Both the calcium load and distribution, as well as the raphe type and length, have been identified as important anatomical characteristics that should be measured in BAV stenosis [48]. According to the relative significance of those morphological characteristics, 0.5–2.0 mm should be detracted from the initial aortic annulus perimeter in order to achieve accurate sizing and the good hemodynamic performance of the transcatheter bioprosthesis. Another method, similar to the previous ones, has been proposed for BAV sizing, with high levels of reproducibility and safety (level of implantation at the RAphe, using LIRA method [49]. According to that, THV prostheses were sized on the basis of baseline CT scan perimeters at the LIRA plane and at the virtual basal ring. In the case of discrepancies between the two plane measurements, the plane with the smallest perimeter was considered the reference for prosthesis sizing.

In the case of severe discrepancies between the measurements and doubt about the correct valve size, the “balloon sizing” method was also proposed: this uses a crude method for estimating the upper diameter of the annulus, where a contrast injection is performed against an inflated balloon in the BAV. In a recent study with a specific type of bioprosthesis (Venus-A Valve, Qiming Medical, Hangzhou, China), the additional sizing of the annulus with balloon pre-dilation prior to implantation in BAV cases resulted in the downsizing of the bioprosthesis [50]. In cases deemed difficult to measure with CT, three-dimensional transesophageal echocardiography (3D-TEE) proved superior to annulus sizing, except for in patients with a severely calcified annulus due to partial acoustic shadowing, which may lead to image inaccuracy [51].

In BAV cases, the meticulous study of the pre-procedural aortic CT anatomy is one of the most important requirements for the selection of the right size and type of bioprosthesis. Among them, calcium distribution in the native BAV has been proven as one of the most important parameters associated with the successful placement of the bioprosthesis. Compared to tricuspid valves, the calcium may differ significantly in BAV cases, and along with the site and extension of the raphes, may result in asymmetrical valve expansion. Apart from the load and distribution of calcium on the BAV, the presence and quantity of calcium in peri-annular areas and the left-ventricular outflow tract (LVOT) are highly associated with PVL, an important factor associated with a poor long-term outcome [52].

## 11. Challenges of TAVI in BAV

BAV-related AS is associated with a greater amount of calcification, mainly due to hormonal, genetical, cellular, inflammatory and possible hemodynamic factors; thus, pre-dilatation with a balloon is more often required during the TAVI procedure [11]. This may lead to a greater rate of PVL and stroke [46]. Fan et al., in 2020, showed that patients with BAV-related AS had a higher risk of stroke when undergoing TAVI, compared to patients with tricuspid valves (2.1% vs. 1.2% during in-hospital stay, 2.5% vs. 1.6% at 30 days post TAVI) [53]. This risk may be reduced using specific embolic protection devices, but since this is not the standard procedure, there must be larger-scale trials to further evaluate the effectiveness of this technique.

BAV is related to a variety of coronary anomalies. This fact must be considered during the selection of patients with BAV-related AS when planning for TAVI. Patients with BAV without the presence of a raphe and with an orifice that is vertically oriented may be at a higher risk of coronary ostia occlusion during TAVI procedure, due to the narrow distance between the two coronary ostia. The coronary occlusion rate in TAVI procedures for BAV-related AS may be as high as 0.1% to 1.2% depending on the study, but there were no differences between SE and BE valves [54,55].

In general, patients with AS may require a pacemaker implantation after a TAVI procedure. This depends on various characteristics, such as the use of a SE valve, the implantation depth and the amount of calcium in the native valve. In patients with BAV-related AS, there may be an even higher rate of pacemaker implantation, attributed mostly to the rather asymmetric expansion of TAVI due to the peculiar anatomy of BAV. The risk is particularly higher in valves with a calcified raphe, since this anatomic characteristic usually drives TAVI expansion towards the conduction system [3,56]. Patients with BAV may experience mixed valvular disease (stenosis—regurgitation), and although they may be eligible for TAVI, there are only limited data in the literature regarding the outcome of these patients. The Society of Thoracic Surgeons/American College of Cardiology transcatheter valve therapy registry showed moderate to severe AR in 3% to 15% of patients finally treated with TAVI. However, there were no specific subgroup analyses for the mixed aortic valve disease cohort [57].

## 12. Technical Considerations in BAV TAVI Implantation

In general, TAVI in BAV patients is a challenging procedure because of the anatomical complexity (coexistence of aortic aneurysm, large annulus, and presence of extreme calcium), the patients’ young age, and the possible need for further interventions in the future (aortic surgery, re-TAVI, etc.). Therefore, special attention is needed during implantation when aiming to achieve the best acute and long-term function (Table 1).

**Figure 2:**
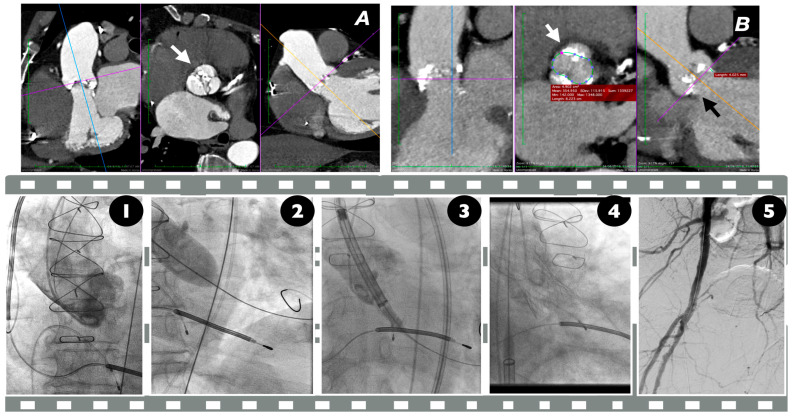
Case illustration of a patient with BAV who underwent TAVI: (**A**) computer tomography angiography (CTA), which reveals typical BAV anatomy Type 3 (white arrow) according to the classification presented in Figure 1; (**B**) the dimensions of the appropriate TAVI size were measured at 4 mm above the aortic annulus. Cinematic representation: 1. baseline angiography, 2. pre-dilatation, 3. SE valve positioning, 4. SE final position and angiography demonstrating no AR, 5. Successful access site closure.

In most of the cases, the bioprosthesis needs to be implanted higher than in tricuspid aortic valve stenosis cases. The presence of raphe in BAV cases usually restricts the circumferential opening of the bioprosthesis. In order to achieve a larger functional area, the bioprosthesis may need to be placed in a higher position. In balloon-expandable bioprosthesis, a typical “80/20” positioning is recommended in most BAV cases. There are, however, specific situations, like large annuli, severe calcification, and a smaller supra-annular space, in which a higher positioning may be required. In those cases, special attention must be paid to avoid valve migration, either upwards or downwards into the ventricle [58]. In SE valves, a higher position is generally the most favorable. In this way, the largest functional area can be achieved and the risk of valve constriction is limited. From this perspective, the “anchoring point” for SE valves should be in the native valve’s cusps, instead of the annulus itself. However, special attention should be paid to allowing enough “anchoring area” for post-dilatation if needed, a procedural step common in BAV TAVI cases (Figure 2—Case Illustration).

**Figure 3:**
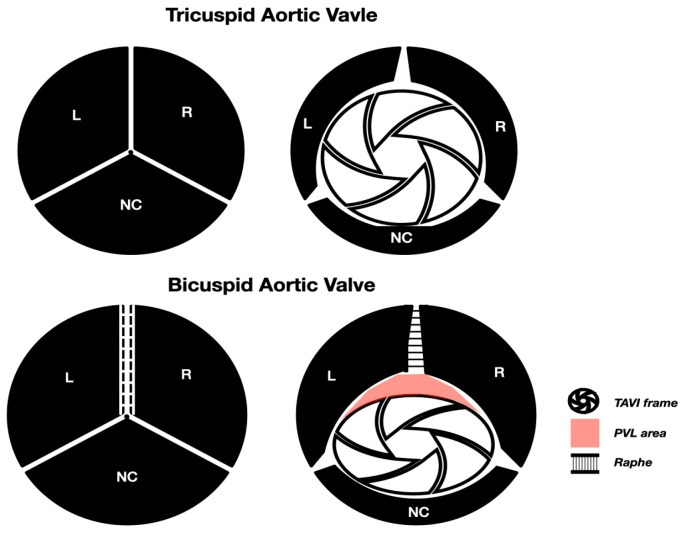
Homogenous and circumferential expansion of a TAVI valve in a tricuspid aortic valve anatomy, which results in a wider distention of the TAVI frame and, subsequently, a larger bio-prosthetic aortic valve area. The homogenous opening of the native valve results in the better apposition of the TAVI valve and better sealing, with less PVL. In a bicuspid anatomy (this is an example of a single raphe BAV, Sievers Type 1 L-R), the presence of a raphe between the cusps may lead to the insufficient separation of the cusps, leading to non-homogeneous TAVI frame expansion. This leads to a smaller valve orifice area and an oval-shaped valve configuration, which may induce early valve degeneration. Furthermore, the incomplete and non-circumferential apposition of the valve’s skirt may lead to incomplete sealing and subsequently a large area for PVL (BAV: Bicuspid Aortic Valve, PVL: Paravalvular leak).

Pre-dilatation with an adequate size balloon is an essential step in TAVI procedures in BAV patients. Even though several randomized and meta-analysis studies have shown that pre-dilatation may be omitted in some tricuspid TAVI procedures, this is not the case for BAV cases, as most of these studies have excluded patients with a BAV anatomy [59,60]. Furthermore, the unique anatomical characteristics of BAV, like the presence of restrictive raphe and/or extreme calcium distribution, make those TAVI procedures challenging and prone to complications (Figure 3). Therefore, pre-dilatation should be strongly considered in all BAV TAVI cases. The selection of the appropriate balloon size for pre-dilatation is important. The diameter of the balloon should be adequate enough to expand and separate the cusps, but within the limits of the annulus to avoid potential rupture. Adequate cusp separation has been shown to offer better bioprosthesis accommodation and expansion reducing rates for valve constriction, subsequent PVL or valve malfunction [61].

Generally, in degenerative tricuspid AS, both balloon and SE valves have demonstrated equal performance in terms of their early functionality and long-term durability. Bicuspid AS shows significant differences in its type, anatomy and characteristics, making each case unique. For example, the presence of fusion between the non-coronary and the right aortic cusp is associated with significant changes in ascending aorta hemodynamics compared to the fusion between the right and left cusp [62]. Subsequently, the type and distribution of calcium also differs. The phenotype of BAV fusion, along with the location of the raphe, is associated with changes in the regional wall shear stress distribution in the aorta and the manifestation of BAV aortopathy, significantly altering the orientation and angulation of the ascending aorta [63]. Therefore, different types of TAVI bioprosthesis may present different performances in different phenotypes of BAV. Data from the large registry BEAT (balloon versus self-expandable valve for the treatment of bicuspid aortic valve stenosis), which included 353 patients, demonstrated an equivalent performance in terms of the overall and cardiovascular mortality rates at 1-year follow-up [64]. There was no difference in the need for a permanent pacemaker, but the BE valves had higher gradients and higher rates of annular rupture. On the other hand, SE valves had higher rates of moderate to severe AR. These results have been confirmed by a later metanalysis, which showed no difference in short- and mid-term mortality, but signaled again the higher number of annular ruptures that occur with balloon-expandable valves [65]. Interestingly, the data showed significant changes in the outflow hemodynamics of the ascending aorta according to the type of bioprosthesis implanted. Patients who were treated with self-expandable valves saw a significant improvement in their helical flow and a reduction in the aortic wall shear stress post TAVI, compared to those who received balloon-expandable valves [66].

Even though both TAVI types are excellent choices for BAV patients, it appears that SE valves may be better with regard to the hemodynamics post TAVI. Whether this translates to the better durability of one valve over the other is not known, as no data are currently available regarding the long-term functionality and performance. Therefore, a tailored approach to selecting the right valve for the right patient, according to the specific BAV phenotype, calcium distribution, type and magnitude of aortopathy, and vascular access anatomy, is the best way to achieve optimal results for TAVI patients with BAV stenosis.

## 13. Gaps in Knowledge

As BAV is considered a combined disease involving the AS and the ascending aorta, considerable attention should be paid to the management of the dilation of the ascending aorta. Until now, all patients with BAV stenosis who undergo the TAVI procedure are treated for only one of the components of the disease. Therefore, it is important to select candidates for whom a possible future aorta intervention would be minimal. Previous reports have shown that the correction of hemodynamics with TAVI in BAV patients may slow or even halt the dilation of the ascending aorta [67]. It has also been shown that initial aortopathy may not affect future interventions in patients undergoing TAVI for BAV [68]. However, these data refer to selected patients who had no indication for any ascending aorta intervention at the time of TAVI.

Stroke is an important complication in BAV patients undergoing TAVI. Data from registries, regarding both self-expandable and balloon-expandable valves, have shown no significant difference between BAV and tricuspid TAVI patients in terms of stroke rates at 30 days and one year [33]. As BAV patients are relatively younger, it has to be stressed that a stroke would have a rather significant impact. Therefore, additional efforts must be made to further reduce the stroke rates in these patients. Data from retrospective studies demonstrated lower in-hospital rates of stroke when using a cerebral protection device, without significantly increasing the hospitalization cost [69,70]. The identification of patients at a high risk of stroke in whom the application of a cerebral protection device would be useful requires supplementary research.

The need for a permanent pacemaker is another problem in TAVI patients. Registries have reported higher rates of pacemaker implantation in BAV patients compared to those with tricuspid AS. Better pre-procedural planning, using detailed CTA information, and the accurate positioning and deployment of the valve have been shown to reduce the need for a permanent pacemaker. Recent data have shown that pacemaker implantation has no significant impact on patients’ prognosis. However, these studies mostly included younger patients with tricuspid AS [71]. No sufficient data exist for BAV patients who have a specific anatomy, younger age and longer life expectancy. It is known that BAV TAVI is associated with a higher incidence of complete heart block and permanent pacemaker insertion, compared to the surgical replacement [38]. Therefore, any long-term interventional strategy for these patients should take into account these limitations. Adequate, larger and more detailed data are needed to evaluate the use of contemporary techniques and devices. Furthermore, since those patients with bicuspid anatomy are excluded from most TAVI trials, in order to validate the use of TAVI in BAV patients as a first option, additional, more robust and sufficient data from larger trials conducted in younger as well as older patients are urgently needed.

**Figure 4:**
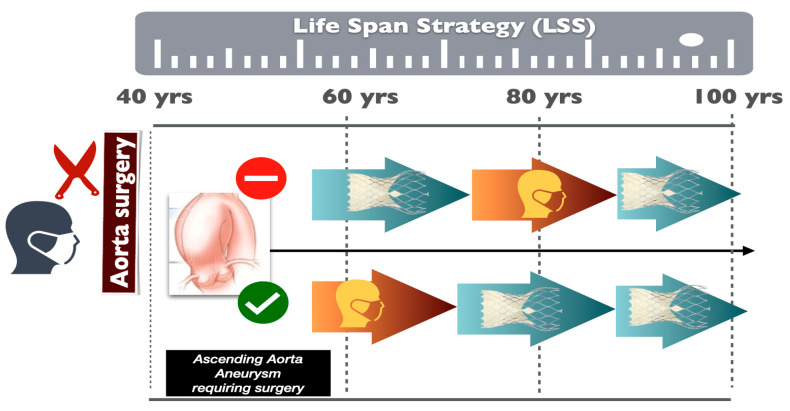
Life Span Strategy (LSS): In patients where surgery is not considered as the only option, due to relative contraindications, deciding how to treat these patients depends on whether the ascending aorta aneurysm requires surgical restoration (✅) or not (⛔). If a surgical correction is required (✅), then the patient may be treated with the initial combined restoration of the ascending aorta and aortic stenosis, and then with TAVI in a bioprosthetic and re-TAVI if needed, at a later stage. In the latter case (⛔), the patient may initially have a TAVI procedure, followed by a surgical combined procedure with ascending aorta and aortic valve replacement in the case of TAVI restenosis and/or ascending aorta aneurysm enlargement. At a later stage, the patient may require a TAVI in a bioprosthetic valve procedure.

One of the most important issues for patients with severe BAV stenosis who are considered candidates for TAVI is long-term holistic management. Apart from AS, those patients suffer from aortopathy which may require future repair. Furthermore, due to their young age, in the presence of coronary artery disease, these patients may need possible coronary interventions. These aspects should be taken into account when deciding to perform TAVI. Even though every patient has their own specific characteristics, a general holistic approach for BAV stenosis patients is proposed in Figure 4; this mainly includes the need for ascending aorta repair and the need for coronary intervention, either with CABG or PCI. However, it has to be emphasized that, especially in young BAV patients, a possible “first-TAVI” approach is not recommended, and it should be only considered in the presence of significant comorbidities or contraindications that exclude surgical replacement.

## 14. Conclusions

Bicuspid AS is a common yet specific phenotype of aortic anatomy, and presents unique characteristics of AS. In severe AS, where the restoration of aortic function is needed, the TAVI procedure may offer excellent results. The choice of the appropriate TAVI bioprosthesis for the right patient, along with the use of modern implantation techniques, can ensure a good outcome with long-term device functionality.

## Figures and Tables

**Table 1:** Special considerations and differences in TAVI procedures between patients with bicuspid and tricuspid severe aortic stenosis.

		Tricuspid	Bicuspid
**Clinical**			
	**Age**	Older	Younger
	**Comorbidities**	More, mostly due to age	Usually specific contraindications to SAVR
**Anatomy**			
	**Structure**	Commonly Tricuspid	Bicuspid with several forms and types
	**Calcification**	Relatively more homogenous	Extensive and asymmetric calcification
	**Presence of Raphe**	No	Presence in one or more cusps
**Pre-Procedural**			
	**Imaging**	Detailed CTA	Detailed CTA
	**Measuring**	Normal measurement dimensions at the level of the annulus	Different level of annulus dimensions according to the anatomy
**Procedural**			
	**Balloon Pre-dilatation**	Usually necessary, but in some cases may be omitted	Usually necessary due to extensive calcification
	**TAVI device Positioning**	Normal positioning to ensure good valve function according to the bioprosthesis type	Occasionally, a higher position is required to achieve larger valve functional orifice
	**Balloon Post-Dilatation**	Not always when the TAVI valve has achieved good expansion and function	Most of the times, it is necessary to adequately expand the valve and increase the valve orifice
**Complications**			
	**Paravalvular Regurgitation**	As expected	Higher rates due to irregular calcification and incomplete valve expansion.
	**Risk of Stroke**	As expected	Higher rates potentially due to more extensive calcification.
	**Pacemaker Implantation**	As expected	Higher rates due to asymmetric expansion of the bioprosthesis

Abbreviations: SAVR: surgical aortic valve replacement, CTA: computer tomography aortography.

## Abbreviations

BAV	Bicuspid Aortic Valve
AS	Aortic Stenosis
AR	Aortic Regurgitation
TAVI	Transcatheter Aortic Valve Implantation
BE	Balloon Expandable
SE	Self-Expandable
